# Influence Mechanisms of Trace Rare-Earth Ce on Texture Development of Non-Oriented Silicon Steel

**DOI:** 10.3390/ma18153493

**Published:** 2025-07-25

**Authors:** Feihu Guo, Yuhao Niu, Bing Fu, Jialong Qiao, Shengtao Qiu

**Affiliations:** 1 School of Metallurgy, Northeastern University, Shenyang 110819, China; guofeihu2020@163.com; 2 Iron and Steel Research Institute Co., Ltd., Beijing 100081, China; niuyuhaoahut@163.com (Y.N.); qiustchina@126.com (S.Q.); 3 Silicon Steel & Sheet Business Division, Xinyu Iron and Steel Group Co., Ltd., Xinyu 338001, China; fubing1986yj@163.com

**Keywords:** rare earth Ce, non-oriented silicon steel, recrystallization kinetics, texture, microstructure

## Abstract

The effects of trace Ce on the microstructure and texture of non-oriented silicon steel during recrystallization and grain growth were examined using X-ray diffraction and electron backscatter diffraction. Additionally, this study focused on investigating the mechanisms by which trace Ce influences the evolution of the {114} <481> and γ-fiber textures. During the recrystallization process, as the recrystallization fraction of annealed sheets increased, the intensity of α-fiber texture decreased, while the intensities of α*-fiber and γ-fiber textures increased. The {111} <112> grains preferentially nucleated in the deformed γ-grains and their grain-boundary regions and tended to form a colony structure with a large amount of nucleation. In addition, the {100} <012> and {114} <481> grains mainly nucleated near the deformed α-grains, which were evenly distributed but found in relatively small quantities. The hindering effect of trace Ce on dislocation motion in cold-rolled sheets results in a 2–7% lower recrystallization ratio for the annealed sheets, compared to conventional annealed sheets. Trace Ce suppresses the nucleation and growth of γ-grains while creating opportunities for α*-grain nucleation. During grain growth, trace Ce reduces γ-grain-boundary migration rate in annealed sheets, providing growth space for {114} <418> grains. Consequently, the content of the corresponding {114} <481> texture increased by 6.4%, while the γ-fiber texture content decreased by 3.6%.

## 1. Introduction

High-grade non-oriented silicon steel, prized for its low iron loss and exceptional cost-effectiveness, serves as an indispensable core material in high-end household appliances, industrial motors, and new-energy vehicle traction motors [1,2]. Against the macro backdrop of “carbon peaking and carbon neutrality” and the pursuit of green, low-carbon development, coupled with the advancement of policies such as power equipment energy-efficiency upgrade standards and the new-energy vehicle (NEV) industry development plan, the demand for high-efficiency non-oriented silicon steel products has significantly increased among users, while higher requirements have been placed on the magnetic properties of the material [3,4,5]. Rare-earth elements, as elements with a unique 4f sublayer electron shell structure and strong chemical activity, are used as important microalloying elements to improve the quality of steel. Rare-earth elements enhance the magnetic properties of non-oriented silicon steel through dual mechanisms of inclusion control and texture optimization. The development of high-quality rare-earth-containing non-oriented silicon steel with distinctive characteristics holds broad application prospects and significant commercial value [6,7,8].

The present industrial production of non-oriented silicon steel by Baosteel can be summarized by noting that the rare-earth content has been controlled at 0.002–0.006%, which could improve the purity of molten steel, spheroidize rare-earth oxide inclusions, and reduce the number of fine inclusions (≤1 μm) [9]. An appropriate amount of rare-earth content in steel can effectively modify inclusions and weaken the influence of inclusions on grain growth and magnetic properties [10,11]. Studies [12,13,14] have shown that trace amounts of rare-earth elements (0.002–0.0051%) could effectively improve magnetic properties. The main reason noted was that rare-earth elements could significantly reduce the hindrances offered by inclusions and precipitates in steel to grain-boundary movement, so that the grain size in the annealed sheet increased, thus effectively inhibiting the nucleation and growth of {111} oriented grains. He et al. [15] studied the effects of rare earth on the microstructure and texture of non-oriented silicon steel. The addition of 0.0051% of a rare-earth element was beneficial to the enhancement of {113} <361> and λ-fiber (<001>//ND), and the unfavorable γ-fiber (<111>//ND) was significantly reduced. On the other hand, excessive rare earth led to the opposite effect.

More importantly, the segregation effect of rare earth as to grain boundaries and the effects on inclusions jointly determine the development of recrystallization texture. Li et al. investigated the preferential occupation behavior of rare-earth Ce and its influence on the electronic structure of Fe at the grain boundary by using the first-principles calculation method and subsequently discussed the grain-boundary segregation behavior of rare earth at the atomic level [16,17]. In summary, a consensus exists that the trace rare-earth treatment of non-oriented silicon steel effectively controls inclusions and enhances magnetic properties. However, the mechanism by which rare-earth element segregation at grain boundaries affects the development of recrystallization textures remains unclear. Specifically, the influence mechanisms of Ce on the nucleation of specific texture grains and grain-boundary mobility have not been established [18,19,20].

In this research, by analyzing the microstructure and texture of non-oriented silicon steel annealed at 600–940 °C, the influence mechanisms of Ce on the recrystallization behavior of cold-rolled sheet and the nucleation and growth of γ-grains and α*-grains are investigated, and then the mechanism of rare earth as it affects the magnetic properties of non-oriented silicon steel is clarified [20,21]. The purpose is to provide a theoretical basis for the application of trace amounts of rare earth in non-oriented silicon steel.

## 2. Materials and Methods

Based on previous experimental results [11,12,13,14,15] associated with rare earth-treated non-oriented silicon steel, the addition of 0.002–0.005 mass% Ce yields optimal magnetic properties. To isolate the effects of trace amounts of rare earths on recrystallization texture, this study selected Ce additions of 0% and 0.0026 mass%. Ce-0 represents the conventional production process, while Ce-26 indicates the rare-earth cerium treatment during the RH refining process. Their main chemical compositions are listed in Table 1. The Ce content in the steel was quantified by ICP-MS, and other components were analyzed using optical emission spectrometry. Both production batches were characterized by identical manufacturing standards and subsequent processing routes. The production process involved soaking a 240 mm thick slab at 1140 °C for 120 min, hot-rolling it to 2.3 mm thickness, normalizing the hot-rolled sheet at 950 °C for 3 min under atmospheric conditions, cold-rolling to 0.30 mm, and finally, continuous annealing at 940 °C to produce the final sheet.

Under identical industrial normalization conditions, the two normalized sheets demonstrated comparable grain sizes. The influence of pre-cold-rolling grain size on annealing recrystallization was negligible. Cut samples (30 mm TD × 300 mm RD) from the cold-rolled sheets underwent annealing experiments in a 70% N_2_ + 30% H_2_ atmosphere furnace: recrystallization annealing at 600–760 °C for 3 min (20 °C increments) and grain growth annealing at 820–940 °C for 3 min (60 °C increments).

Annealed specimens from the above experiment, after standard metallographic grinding and polishing, were etched with 4% nital solution for microstructural and microtextural characterization. Metallographic structures were observed under optical microscopy (OM), and the recrystallized fraction was estimated by measuring the area percentage of recrystallized grains in the annealed sheets, using Image-Pro 6.0 software [22]. The micro-texture was detected by a scanning electron microscope (ZEISS SUPRA 55VP, Jena, Germany) equipped with an EDAX OIM electron backscatter diffraction (EBSD) system, operating at 20 kV with a scan step size below 1/10 of the average grain diameter. The orientation distribution function (ODF), the distribution and quantitative statistics of specific orientation grains, and the grain-boundary angle were analyzed by OIM Analysis 7.3 software. The main texture content was calculated with a deviation angle of 15°. To ensure the statistical significance of the detected microtextural data, a minimum of 400 grains were characterized per specimen. Macro-texture of annealed sheets was characterized using an X-ray diffractometer (PANalytical EMPYREAN SERIES 2, Almelo, The Netherlands) with Co-Kα radiation at 35 kV and 40 mA. Acquired diffraction data were processed through X’Pert Texture 1.2 software for Orientation Distribution Function (ODF) computation.

## 3. Results and Discussion

### 3.1. Recrystallization Process Analysis

**Figure 1:**
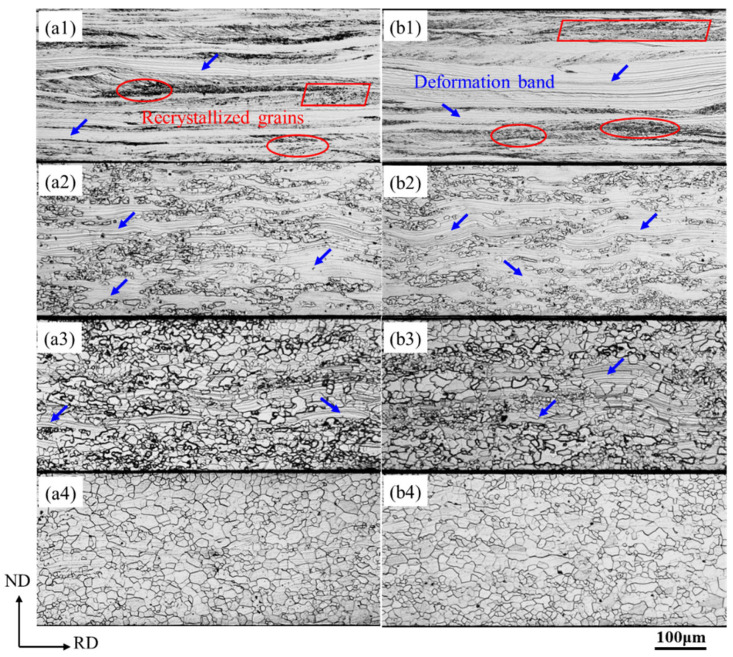
Microstructures of Ce-0 and Ce-26 after annealing at different temperatures. (**a1**,**b1**) 640 °C, (**a2**,**b2**) 680 °C, (**a3**,**b3**) 720 °C, and (**a4**,**b4**) 760 °C; Ce-0 (**a1**–**a4**), Ce-26 (**b1**–**b4**).

**Figure 2:**
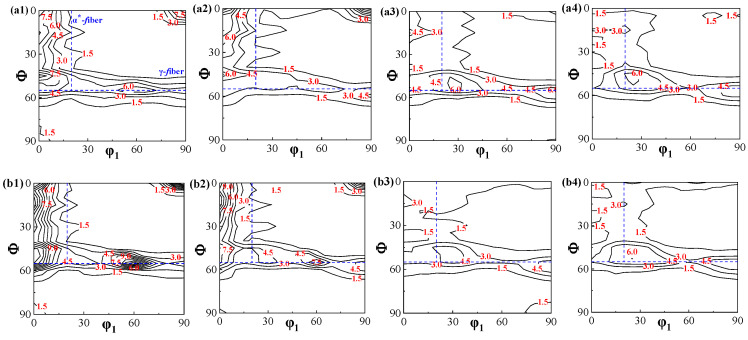
Macro-textures (φ_2_ = 45° ODF section) of Ce-0 and Ce-26 after different annealing temperatures. (**a1**,**b1**) 640 °C, (**a2**,**b2**) 680 °C, (**a3**,**b3**) 720 °C, and (**a4**,**b4**) 760 °C; Ce-0 (**a1**–**a4**), Ce-26 (**b1**–**b4**).

The microstructure and macro-texture of the Ce-0 and Ce-26 cold-rolled sheets after annealing at 640 °C, 680 °C, 720 °C, and 760 °C are shown in Figure 1 and Figure 2. It can be seen that after annealing at 640 °C for 3 min, the annealed sheet is still dominated by deformation band structure, and there are small amounts of recrystallized grains at the grain boundaries of shear bands and deformed grains. The texture consists of strong α-fiber and weak γ-fiber textures, with the maximum intensity at {100} <110> and {111} <110> components. With the increase in annealing temperature, the deformation structure gradually decreases due to the increase in the recrystallization ratio. The α-fiber intensity in the annealed sheet decreases gradually, while the α*-fiber intensity increases. Meanwhile, the γ-fiber texture gradually dominates, and the maximum intensity shifts from the {111} <110> component to the {111} <112> component. After annealing at 760 °C for 3 min, the annealed sheet is completely recrystallized, and the cold-rolled deformed structure is replaced by recrystallized grains. Therefore, the strong γ-fiber with a maximum intensity at the {111} <112> component and the weak α*-fiber with a maximum intensity at the {114} <481> component are observed.

The Arrhenius formula is generally used to describe the effect of annealing temperature (*T*) on recrystallization rate (*v*) in the study of recrystallization kinetics of non-oriented silicon steel during annealing. Under the same annealing time (*t*), the recrystallization ratio (*f*) of the annealed sheet is proportional to the recrystallization rate. The recrystallization rate (*v*) and its relationship with the recrystallization ratio (*f*) are shown in Formulas (1) and (2).

(1) $$v=Ae^{-Q/RT}$$

(2) $$v\propto f=A\prime e^{-Q/RT}$$

where *A* and *A*′ are constants, respectively; *Q* is the recrystallization activation energy; *R* is the gas constant.

**Figure 3:**
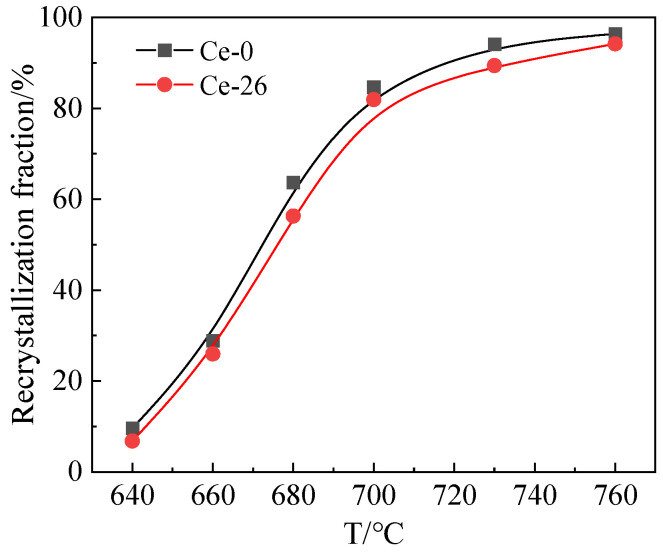
Relationship between recrystallization fraction and annealing temperature, for Ce-0 and Ce-26.

Figure 3 illustrates the recrystallization fraction trends of the Ce-0 and Ce-26 annealed sheets versus annealing temperature. The data reveal that recrystallization percentages increase with rising annealing temperatures. Under identical annealing conditions, the recrystallization initiation temperature of Ce-26 is higher than that of Ce-0, resulting in a recrystallization rate 2–7% higher for Ce-0 compared to Ce-26. Ce atoms (*r_Ce_* ≈ 0.182 nm) exhibit a significant atomic size mismatch δ (δ ≈ 48%) with matrix atoms (*r_Fe_* ≈ 0.124 nm). According to the Fleischer–Labusch solid solution strengthening theory [23], the elastic stress field generated by the misfitting atoms interacts with the dislocation strain field, thereby increasing the dislocation slip resistance. The model established based on the quasiparticle (QA) method of atomic density functional theory explains the interaction between the solute atoms and dislocation configurations: the rare-earth element Ce can form a Cottrell (solute) atmosphere around the dislocation core [24,25,26]. The solute Ce is thought to influence dislocation motion during recovery via the solute drag effect. Solute segregation around the dislocation core creates a pinning effect, immobilizing the dislocation. Under these conditions, the dislocation must overcome a higher driving pressure to escape the solute atom environment. This leads to the requirement for a higher recrystallization activation energy to initiate the recrystallization process [27]. This theoretically demonstrates that rare-earth elements exert an inhibitory effect on overall recrystallization.

### 3.2. Texture Evolution During Recrystallization

**Figure 4:**
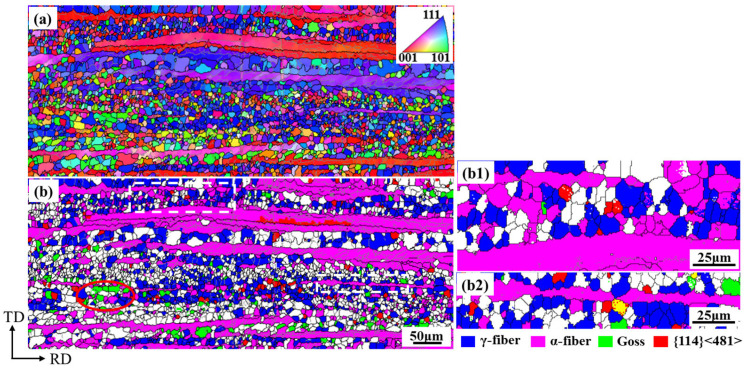

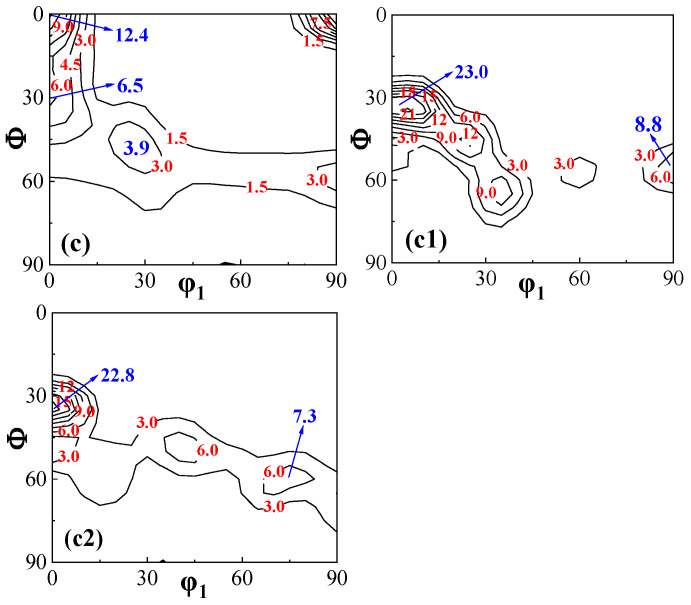
Orientation image maps of Ce-0 cold-rolled sheet annealed at 680 °C for 3 min. (**a**) grain orientation image maps; (**b**,**c**) (**b1**,**c1**), (**b2**,**c2**) several main-texture distribution maps and corresponding ODFs at φ_2_ = 45° section.

**Figure 5:**
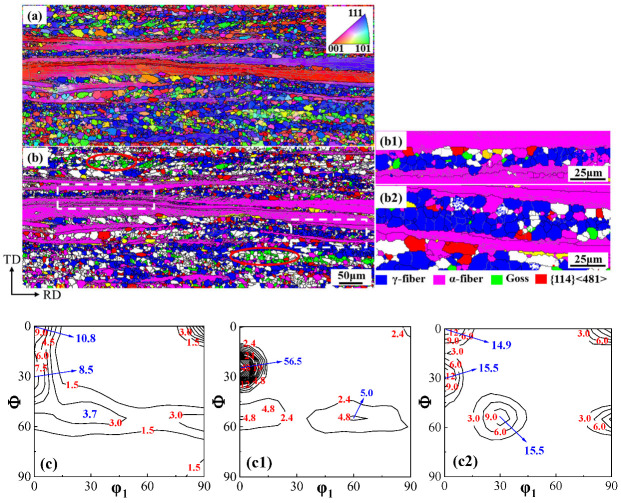
Orientation image maps of Ce-26 cold-rolled sheet annealed at 680 °C for 3 min. (**a**) grain orientation image maps; (**b**,**c**) (**b1**,**c1**), (**b2**,**c2**) several main textures distribution maps and corresponding ODFs at φ_2_ = 45° section.

During the recrystallization process of non-oriented silicon steel, the newly nucleated grains have a specific orientation relationship with the deformed microstructure, which determines the recrystallization texture. As shown in Figure 1, partial recrystallization occurs in both Ce-0 and Ce-26 cold-rolled sheets under the annealing conditions of 680 °C × 3 min. Combined analysis of the specific texture distributions in Figure 4b and Figure 5b with their corresponding ODFs reveals that the annealed sheets are mainly composed of the deformed structure of α-fiber texture extending along the rolling direction, in addition to the recrystallized grains dominated by γ-fiber texture and a small amount of Goss and α*-fiber textures.

During cold rolling, matrixes with different orientations exhibit distinct deformation rates. The more easily deformed the matrix, the lower its stored energy. Along the α-fiber, the stored energy of the deformed grains follows the following order: {100} < {112} < {111} < {110} [28]. During the annealing process, Goss and γ-fiber textures recrystallize first due to high energy storage. Since the smaller {114} <481> grains mainly exist in the {112} <110> deformed grains, the {114} <481> recrystallized grains mainly nucleate and grow near the grain boundaries of the deformed α grains at the initial stage of recrystallization. However, as a stable rolling texture, the α-fiber texture is difficult to recrystallize due to the low dislocation density, especially {100} <110> texture [29,30]. Therefore, in the early stage of the recrystallization of non-oriented silicon steel, due to the high energy storage and dislocation density in the deformed grain and grain-boundary region of γ-fiber texture, the preferential nucleation position for {111} <112> oriented grains is provided, and the recrystallized γ-grains tend to form colony structures. The recrystallized Goss grains mainly nucleate at the shear band position in the deformed γ-grains and are mainly adjacent to the recrystallized γ-grains after recrystallization (as shown in the red circles in Figure 4b and Figure 5b). The nucleation density of {100} <012> and {114} <481> recrystallized grains is limited, and primarily localized adjacent to deformed α-grains.

To analyze the effect of Ce on the growth of recrystallized grains within the deformed microstructure, characteristics of recrystallized γ-grains and adjacent non-recrystallized deformed α- grains in the micro-regions of Figure 4b and Figure 5b were compared. In the Ce-0 annealed sheet exhibiting a high recrystallization degree, γ-grains grew in the regions of Figure 4(b1,b2), with an average grain size of 9.4 μm, consuming the adjacent deformed α-grains. As shown in Figure 5(b1,b2), recrystallization was delayed in the Ce-26 annealed sheet. Recrystallized γ-grains existed among coarse deformed α-grains, exhibiting a smaller average grain size of 7.7 μm and reduced consumption of deformed α-grains.

**Figure 6:**
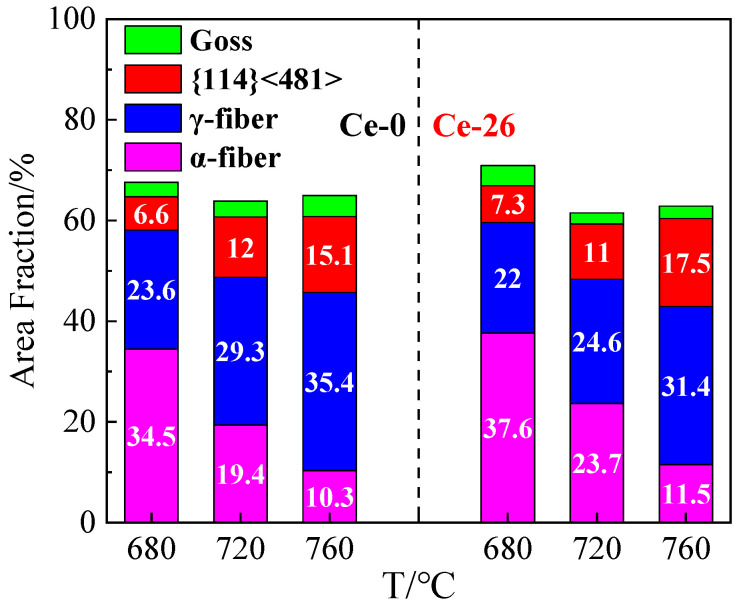
The area fractions of specific textures in the recrystallization processes of Ce-0 and Ce-26 cold-rolled sheets.

Figure 6 illustrates the variation trends of specific texture components in Ce-0 and Ce-26 cold-rolled sheets after annealing at different temperatures (680 °C, 720 °C, and 760 °C for 3 min). Combined with the annealed sheet microstructures in Figure 1, increasing annealing temperatures promote further recrystallization and grain growth within the deformed structure, enhancing overall recrystallization. During the initial recrystallization stage (680 °C × 3 min), recrystallized γ-grains demonstrate numerical superiority due to advantages in stored energy and nucleation sites, with contents reaching 23.6% in Ce-0 and 22.0% in Ce-26. Conversely, α*-grains exhibit constrained nucleation availability, resulting in lower contents of {114} <481> texture: 6.6% in Ce-0 versus 7.3% in Ce-26.

During recrystallization (760 °C × 3 min), recrystallized grains grow by consuming neighboring deformed α-grains with low stored energy. This results in a significant decrease in contents of α-fiber texture, while γ-fiber and {114} <481> textures increase, with γ-fiber texture being notably more prevalent than {114} <481>. At identical annealing temperatures, the Ce-26 annealed sheets exhibit higher contents of α-fiber texture than Ce-0 sheets, further demonstrating trace Ce’s inhibition of recrystallization. Specifically, the content of γ-fiber texture in Ce-26 sheets is 4.0% lower than in Ce-0, while the content of {114} <481> is 2.4% higher.

In summary, the trace Ce in the non-oriented silicon steel delays the recrystallization process of the annealed sheet, which can effectively inhibit the recrystallization behavior and grain growth of the γ-grains and hinder the swallowing of the deformed α-grains by the γ-grains, providing an opportunity for the recrystallization and grain growth of the α*-grains.

### 3.3. Texture Evolution During Grain Growth Process

**Figure 7:**
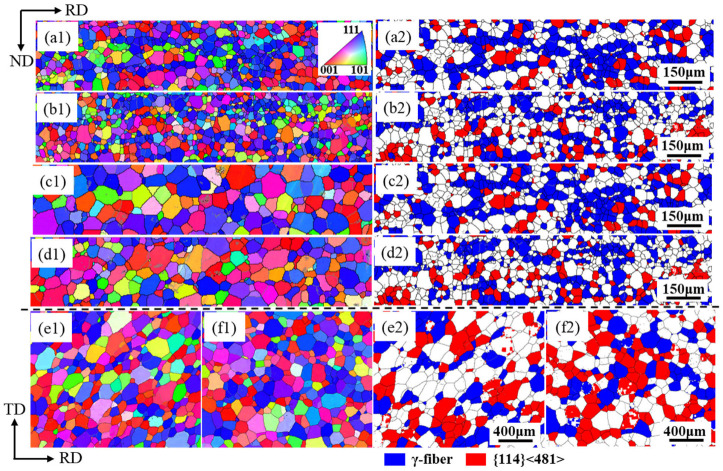
Grain orientation image maps (left) and several typical textures (right) of Ce-0 and Ce-26 cold-rolled sheets under different annealing conditions. (**a**,**b**) 820 °C, (**c**,**d**) 880 °C, and (**e**,**f**) 940 °C; Ce-0 (**a**,**c**,**e**), Ce-26 (**b**,**d**,**f**).

**Figure 8:**
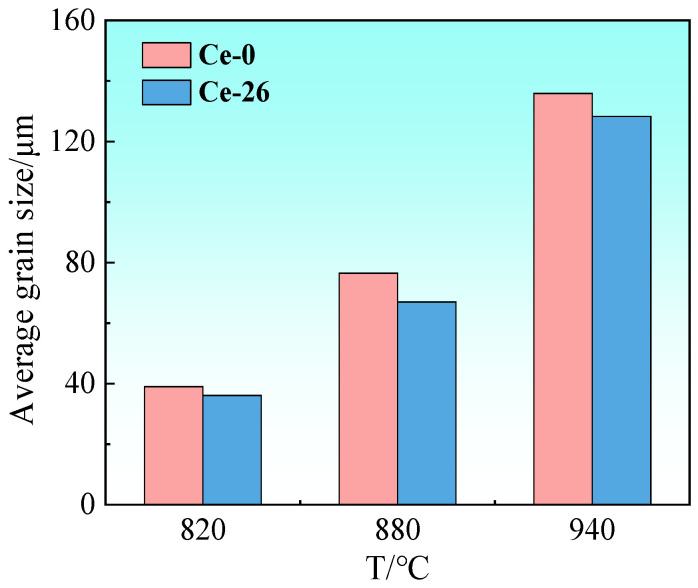
Average grain size distribution for Ce-0 and Ce-26 cold-rolled sheets annealed at different temperatures.

Figure 7 presents grain orientation imaging and specific texture distributions in Ce-0 and Ce-26 cold-rolled sheets annealed at 820 °C, 880 °C, or 940 °C for 3 min. Beyond 820 °C annealing, both sheets undergo complete recrystallization, with textures predominantly consisting of {114} <481> and {111} <112>. Figure 8 shows the trends of average grain size for the Ce-0 and Ce-26 cold-rolled sheets after annealing at 820 °C, 880 °C, or 940 °C for 3 min, indicating that the average grain size increases with the increase in annealing temperature. The average grain sizes of Ce-0 annealed sheets after annealing at 820 °C, 880 °C, or 940 °C for 3 min are 39.0 μm, 76.5 μm, and 135.9 μm, and those of Ce-26 annealed sheets are 36.1 μm, 67.0 μm, and 128.3 μm, respectively. Previous studies [17,31] have demonstrated that first-principles calculations based on density functional theory (DFT) indicate Ce atoms tend to segregate at grain boundaries, at which the system adopts a minimum-energy configuration with enhanced structural stability. Theoretical analysis using the Cahn–Lüicke–Stüwe (CLS) model [18,32] elucidates the rare-earth effects through two mechanisms: grain-boundary segregation and solute drag effects. The segregation of solute Ce at grain boundaries induces severe lattice distortion in the matrix, which exerts a drag force on grain-boundary migration, significantly reducing grain-boundary mobility. This provides a mechanistic explanation for the finer grain size observed in Ce-26 annealed sheets compared to reference materials under identical annealing conditions.

**Figure 9:**
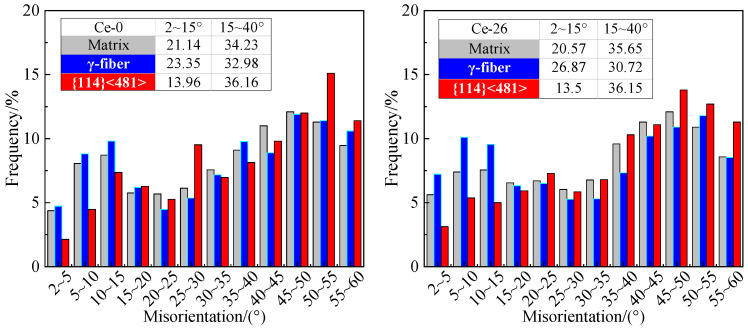
Distribution of orientation deviation between specific orientation grains and the adjacent grains in Ce-0 and Ce-26 annealed sheets after annealing at 820 °C for 3 min.

Large-angle grain boundaries (15° ≤ θ < 40°) exhibit higher mobility, which is conducive to grain growth, whereas low-angle grain boundaries (θ < 15°) display lower mobility, which impedes growth. The growth tendency of specific grains was determined by comparing adjacent grain-boundary characteristics [33,34]. Figure 9 displays the misorientation angle distributions between the matrix and primary oriented grains in Ce-0 and Ce-26 annealed sheets under 820 °C × 3 min annealing. For {114} <481> grains, the frequency of adjacent low-angle grain boundaries (θ < 15°) is reduced by 7.18% in Ce-0 and 7.07% in Ce-26 compared to the matrix, while both exhibit substantially higher frequencies of high-angle boundaries (15° ≤ θ < 40°) with minimal variation between them. Consequently, {114} <481> grains demonstrate preferential growth in annealed sheets due to their enhanced grain-boundary migration rates. Conversely, γ grains in Ce-0 and Ce-26 sheets show increased low-angle boundary frequencies (up +2.21% and +6.3% versus matrix) and decreased high-angle boundary frequencies (down −1.25% and −4.93% versus matrix). These findings confirm that during grain growth, trace cerium impedes γ-grain development by reducing boundary migration velocity, thereby liberating growth space for preferential expansion of {114} <481> grains.

**Figure 10:**
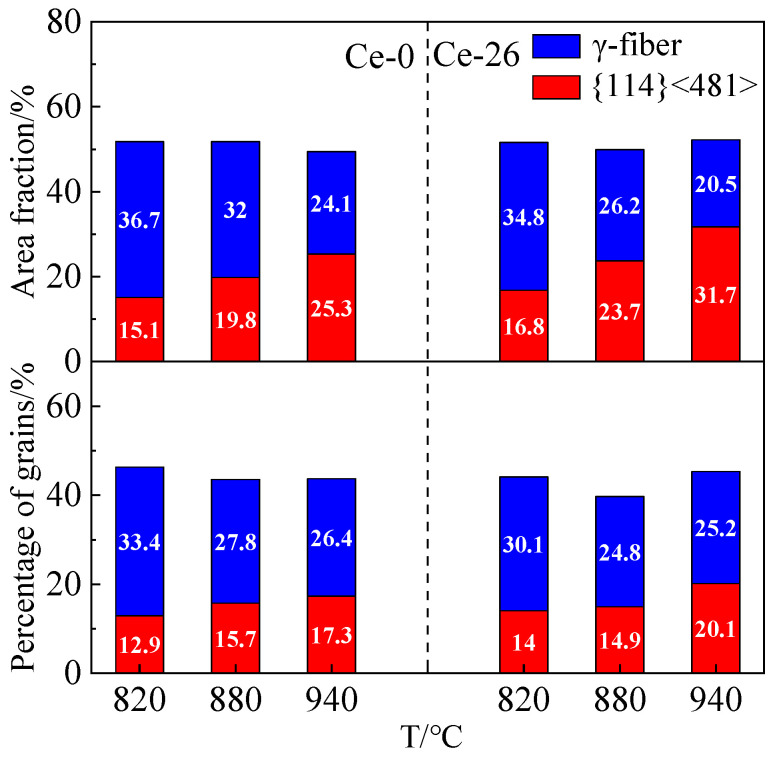
Distributions of specific textures during grain growth in Ce-0 and Ce-26.

To investigate texture evolution during grain growth in annealed sheets, Figure 10 quantifies the content of {114} <481> and γ-fiber textures, along with their corresponding grain proportions at different annealing temperatures. Following 820 °C annealing, the number densities of {114} <481> and γ-grains were measured at 214 and 553 grains/mm^2^ for Ce-0 and 329 and 705 grains/mm^2^ for Ce-26, respectively. This demonstrates that both the number density and proportion of {114} <481> grains in the Ce-26 sheet exceed the corresponding values in the Ce-0 sheet. The γ-fiber texture becomes the dominant type in both Ce-0 and Ce-26 sheets (with contents of 36.7% and 34.8%, respectively), due to its nucleation density advantage during recrystallization. As annealing temperature increases, the {114} <481> grains with high grain-boundary mobility undergo rapid growth, leading to a significant rise in their texture content. Following 940 °C annealing, the content of {114} <481> texture reaches 25.3% in Ce-0 and 31.7% in Ce-26 samples, replacing γ-fiber texture as the dominant component. Meanwhile, γ-fiber texture experiences growth suppression due to its grain-boundary mobility being lower than the matrix average, resulting in reduced contents of 24.1% and 20.5%, respectively. Furthermore, the presence of cerium additionally impedes γ-grain growth, leading to a greater reduction of γ-fiber texture alongside a substantial enhancement in {114} <481> texture development in Ce-26 annealed sheets.

The recrystallization texture of non-oriented silicon steel is determined by the competitive evolution among differently oriented textures during the nucleation and growth stages. Studies [35,36,37] demonstrate that optimizing recrystallization texture represents an effective approach for the enhancement of magnetic properties. This involves increasing the content of the ideal, easily magnetized λ-fiber texture while reducing the detrimental, hard-to-magnetize γ-fiber texture. The {114} <481> texture, being closer to the λ texture, is defined as a favorable texture. Trace cerium suppresses recrystallization behavior by reducing nucleation of γ-grains while simultaneously impeding their growth through decreased grain-boundary migration velocity. Consequently, in the trace rare-earth Ce annealed sheet, the content of {114} <481> texture is higher, while the content of {111} <112> texture is lower, with the resultant texture optimization enhancing magnetic properties in final sheets. Under industrial production conditions, the core loss (P_1.5/50_) values of the finished Ce-0 and Ce-26 sheets are 2.33 W/kg and 2.27 W/kg, respectively, and the magnetic induction (B_50_) values are 1.646 T and 1.651 T, respectively. The magnetic properties of Ce- treated non-oriented electrical steel meet the requirements for commercial grade 30W230.

## 4. Conclusions

(1) Non-oriented silicon steel cold-rolled sheets exhibit a predominant α-fiber texture, with weaker γ-fiber texture. As recrystallization progresses, the intensity of α-fiber texture progressively diminishes, while both α∗-fiber and γ-fiber textures strengthen.
(2) The {111} <112> recrystallized grains preferentially nucleate at the deformed γ-grains and grain-boundary regions and tend to form colony structures. The {100} <012> and {114} <481> recrystallized grains are mainly dispersed near the deformed α-grains.
(3) At a Ce content of 0.0026%, the recrystallization of the cold-rolled sheet is retarded, inhibiting the consumption of deformed α-grains by recrystallized γ-grains, which provides favorable conditions for the nucleation and growth of α*-grains.
(4) During grain growth, {114} <481> grains exhibit accelerated expansion due to their superior grain-boundary migration rates. Meanwhile, trace cerium segregates at grain boundaries and generates solute drag effects, suppressing γ-grain development by reducing boundary mobility.

## Figures and Tables

**Table 1:** Main chemical composition of non-oriented silicon steel (wt %).

Samples	C	Mn	S	P	Si	Al	N	Ce
Ce-0	0.0024	0.27	0.002	0.012	3~3.3	0.8~1	0.0020	0
Ce-26	0.0024	0.29	0.002	0.015	3~3.3	0.8~1	0.0020	0.0026

## Data Availability

The original contributions presented in this study are included in the article. Further inquiries can be directed to the corresponding author.
